# Efficacy and Safety of Teriparatide in Beta-Thalassemia Major Associated Osteoporosis: A Real-Life Experience

**DOI:** 10.1007/s00223-022-00963-3

**Published:** 2022-03-04

**Authors:** Irene Gagliardi, Mariella Celico, Maria Rita Gamberini, Margherita Pontrelli, Monica Fortini, Aldo Carnevale, Nicola Napoli, Maria Chiara Zatelli, Maria Rosaria Ambrosio

**Affiliations:** 1 Section of Endocrinology, Geriatrics & Internal Medicine, Dept of Medical Sciences, University of Ferrara, Via Fossato di Mortara 64/B, 44121 Ferrara, Italy; 2 Unit of Thalassaemia and Haemoglobinopathies Day Hospital, Regional HUB Centre, Department of Medicine, Azienda Ospedaliero Universitaria S. Anna, Cona, Ferrara, Italy; 3 Department of Interventional and Diagnostic Radiology, Arcispedale Sant’Anna, Ferrara, Italy; 4 Division of Endocrinology and Diabetes, University Campus Bio-Medico di Roma, Rome, Italy

**Keywords:** Beta-Thalassemia, Osteoporosis, Teriparatide, Fractures, Bone pain

## Abstract

Osteoporosis represents a relevant cause of morbidity in adult Thalassemia Major (TM) population. Antiresorptive drugs such as bisphosphonates were demonstrated effective in preventing bone loss. Teriparatide (TP) is an anabolic agent approved for osteoporosis management in the general population, but its use has been very limited in TM patients so far. We evaluated TP efficacy and safety in TM-associated osteoporosis in real-life clinical practice. Retrospective evaluation of 11 TM patients (6 males, 5 females; mean age = 45 ± 4.38 years) with severe osteoporosis and multiple fractures under TP treatment. Mean TP treatment duration was 19 ± 7 months. TP withdrawal was due to poor compliance and side effects (fever and osteo-muscular pain) in two and three patients, respectively. After 12 and 24 months, BMD significantly increased at lumbar (+ 19% and 22%) and femoral sites (+ 13% and 13%). Osteocalcin and cross-laps levels increased after 12 and 24 months (+ 225 and + 54.2%; + 159 and 141%, respectively). No new fractures were detected during TP treatment. Baseline VAS score values (3 ± 3) did not significantly change after 12 and 24 months (3 ± 3 and 2 ± 3, respectively). Five out of eleven patients developed side effects. TP might be an effective treatment for TM-associated osteoporosis since it improves BMD, especially at the lumbar spine, and prevents fragility fractures. TM patients may have a higher frequency of side effects, especially muscle and bone pain under TP treatment, as compared to no TM population. Further studies are needed.

## Introduction

Thalassemia Major (TM) is a hereditary hematological disease characterized by a reduced or absent synthesis of β-globin chains. Consequently, unpaired alpha chains precipitate in red cell precursors leading to defective erythroid maturation, ineffective erythropoiesis and peripheral hemolysis. The resulting anemia stimulates bone marrow to an ineffective proliferation which causes bone marrow expansion and consequent skeletal and growth abnormalities [1]. TM chronic anemia usually needs a life-long blood transfusion supply. Improvements in transfusion protocols and implementation of iron chelation therapies have significantly increased TM patients' survival. Subsequently, the increasing life expectancy arises concerns about the development of several systemic comorbidities, such as osteoporosis and fragility fractures, that represent a relevant cause of morbidity [2].

Osteoporosis prevalence in TM ranges from 50.7 to 74.1% at lumbar spine (LS) and from 10.8 to 37.9% at femur site. Fracture prevalence ranges from 12.1 to 35.1%, especially at extremities. Vertebral fractures, instead, are documented as less common, even if they are usually underestimated [3–6]. In addition, bone pain is often reported especially at lower back site, although it is not always due to osteoporosis and bone fractures [7].

The pathogenesis of TM-associated osteoporosis is multifactorial. Bone marrow expansion, iron overload, endocrine complications of TM (especially hypogonadism), chronic liver disease, renal dysfunction, iron chelation therapy (mainly deferoxamine), vitamin D (25(OH)D) and zinc deficiency have been identified as concurrent factors in the development of TM-associated osteoporosis. They induce an imbalanced bone turnover with an increased osteoclast activity and a decreased osteoblast function, leading to suboptimal peak bone mass and a gradual decline in bone mass [4, 8, 9].

The primary approach is the careful management of TM and its complications. Despite proper blood transfusion supply, effective chelation therapy and adequate hormonal replacement therapy, TM patients tend to lose bone mass [10]. The observation of increased bone resorption in TM-associated osteoporosis has led to the use of antiresorptive agents such as bisphosphonates (BP), namely alendronate, neridronate and zoledronate. Even if the number of studies is limited, BP were shown to prevent bone loss, to improve bone mineral density (BMD) and to be well tolerated. However, BP anti-fracture efficacy and safety are not well established, due to the short follow-up and small sample size of trials conducted so far [11]. On the other side, Teriparatide (TP) is the only anabolic therapy approved in Italy for the management of osteoporosis in the general population and is demonstrated as an effective treatment for post-menopausal, male and glucocorticoid-induced osteoporosis, increasing BMD and reducing fracture risk, especially at vertebral sites [12, 13]. In TM patients, TP might address the anabolic bone impairment by inducing osteoblast lining cells differentiation, stimulating osteoblast activity and limiting osteoblast and osteocyte apoptosis [14, 15]. To our knowledge, there are only two case reports published about TP treatment in TM patients. In both these reports, TP treatment improved BMD, was well-tolerated and safe [12, 13]. However, the clinical use of TP in TM-associated osteoporosis has been very limited so far, and more data addressing anti-osteoporotic agents and sequential treatments in TM are strongly warranted due to the need of lifelong management of TM-associated osteoporosis.

Our study aimed at evaluating the efficacy and safety of TP in TM-associated osteoporosis in real-life clinical practice, describing a series of eleven TM patients with severe osteoporosis and multiple fractures.

## Material and Methods

### Patients

We retrospectively evaluated 11 TM adult patients (> 18 years old) affected by osteoporosis on TP treatment, referring to Ferrara Endocrinological and Thalassemic outpatients clinics between 2010 and 2020. We excluded patients with severe kidney, liver or cardiac disease; history of neoplastic disease; history of bone marrow transplantation; pregnancy or breastfeeding.

All patients were treated with subcutaneous TP 20 µg/day for a maximum of 24 months, in agreement with the indications of the Italian Drug Agency (AIFA). According to AIFA guidance, patients received TP prescription if: (1) new moderate-severe vertebral or femoral fracture occurred despite BP therapy; (2) at least three severe vertebral fractures or femoral fracture/2 severe vertebral fractures and 1 femoral fracture occurred in patients naïve for anti-osteoporotic treatment.

All patients were on iron-chelation therapy, and they were regularly blood transfused. In addition, all of them received 25(OH)D and calcium supplementation.

Six out of 11 patients completed 24 months of TP treatment, and the others stopped therapy earlier (3 patients after 12 months, 1 patient after 8 and 1 patient after 6 months) due to adverse events or poor compliance.

This study has been approved by the Local Ethics Committee (Comitato Etico Indipendente di Area Vasta Emilia Centro, CE-AVEC, at the Policlinico S.Orsola-Malpighi in Bologna) and authorized by the General Director of the Azienda Ospedaliero Universitaria in Ferrara (protocol number CE-AVEC 697/2020/Oss/AOUFe). All enrolled subjects read and signed the informed consent form before enrolling in the study.

### Study Design and Methods

We collected baseline data referring to the time before TP treatment: body mass index (BMI); smoke habit; previous anti-osteoporotic therapy (type, duration and washout period); transfusion regimen (pre-transfusion hemoglobin mean annual level; transferrin soluble receptor and degree of erythroid proliferation calculated according to the formula “transferrin soluble receptor/mean value of the reference range” [16]; type of chelation therapy); iron overload markers (ferritin mean annual level; cardiac iron concentration—CIC, T2* ms -, liver iron concentration, mg/g). The T2* technique was used for iron overload assessment. Liver iron concentration (LIC) was calculated from the formula: LIC = (25.4/T2*) + 0.202. In addition, data regarding endocrine diseases (hypogonadism; hypothyroidism; hypoparathyroidism; adrenal insufficiency; growth hormone deficiency; diabetes mellitus) and zinc plasma levels were collected.

For each patient, clinical, biochemical and radiological parameters were assessed at baseline and after 12 and 24 months of therapy. Efficacy and safety outcome was investigated.

Clinical evaluation consisted of assessment of side effects and clinical fractures. In addition, bone pain was quantified through the visual-analogic scale (VAS: 0 = no pain; 10 = worst pain).

Biochemical evaluation included serum calcium, phosphates, bone turn-over markers (osteocalcin, beta-crosslaps and alkaline phosphatase), 25(OH)D and 24 h-urinary calcium and phosphates levels.

Serum/urinary calcium was assessed with a colorimetric/photometric assay, serum/urinary phosphates with photometric UV assay; chemiluminescence assays were used for 25(OH)D (LIAISON© 25 OH Vitamin D TOTAL Assay, DiaSorin), serum/urinary cortisol (Access Immunoassay system, Beckman Coulter), beta-crosslaps (IDS-iSYS CTX-I (CrossLaps®, ids®) and osteocalcin (LIAISON® BAP OSTASE®, DiaSorin); alkaline phosphatase was measured by kinetic colorimetric assay.

Bone mineral density (BMD) was assessed at lumbar spine (LS), femoral neck (FN) and total hip (TH) by dual-energy X-ray absorptiometry (DXA). All patients underwent DXA in the same medical center and were tested with the same Hologic densitometer. We considered absolute BMD values expressed as g/cm^2^ to evaluate TP efficacy outcome. The densitometer in use demonstrated a Least Significance Change (LSC) for lumbar spine, femoral neck and total hip of 0.002 g/cm^2^, 0.029 g/cm^2^ and 0.027 g/cm^2^, respectively. The long-term precision error was ± 1.5%. Vertebral fractures incidence was investigated by performing radiographic morphometric scan. Genant method was applied to assess vertebral fractures. Radiological assessment of femoral and rib fractures was performed only if femoral or rib fractures were suspected. The same expert radiologist re-evaluated both DXA and vertebral radiography.

## Statistical Analysis

Categorical and continuous variables were presented as absolute values/percentages and mean values ± standard deviation (SD), respectively. Wilcoxon test for paired samples was used to evaluate differences between variables at baseline and during TP treatment. Significant differences were defined for *p* values < 0.05. Statistical analysis was performed using the MedCalc software.

## Results

### Patients Characteristics

Baseline clinical characteristics of the 11 recruited TM patients (6 male, 5 female; mean age = 45 ± 4.38 years old) are shown in Table 1. 64% of them had been previously on BP therapy (mean duration treatment of 5.43 ± 0.79 years with a subsequent mean drug holiday of 1.29 ± 0.76 years). Nobody was receiving ongoing antiresorptive therapy.

**Table 1:** Characteristics of subjects before starting teriparatide treatment

Subjects												
	n°1	n°2	n°3	n°4	n°5	n°6	n°7	n°8	n°9	n°10	n°11	TOT
Sex (M/F)	M	M	F	M	M	F	F	F	M	F	M	6 M/5F
Age (years)	44	45	44	45	48	38	37	48	48	52	46	Median (range) 45 (37–52)
Body mass index (kg/m^2^)	16.5	25.5	24.9	25.7	22.0	23.9	18.8	22.8	20.7	21.7	22.9	Median (range) 22.8 (16.5–25.7)
Smoker (Yes/No)	No	No	No	No	No	No	No	No	No	No	No	11 No
Previous bisphosponates therapy (Yes/No)	No	Yes	Yes	Yes	Yes	No	Yes	Yes	No	Yes	No	7 Yes/4 No
Duration previous therapy (years)	–	5	5	6	6	–	6	4	–	6	–	Median (range) 6 (4–6)
Duration drug holiday (years)	–	3	1	1	1	–	1	1	–	1	–	Median (range) 1 (1–3)
Pre-transfusional hemoglobin (g/dl)	8.3	9.3	9.5	10.2	9.7	9.3	9.6	9.7	10.1	9.7	10.3	Median (range) 9.7 (8.3–10.2)
Soluble transferrin receptor (mg/l)	7.9	11.0	12.6	12.2	6.9	16.2	11.7	5.5	14.1	11.5	3.7	Median (range) 11.5 (3.7–16.3)
Degree of erythroid proliferation	2.20	3.06	3.52	3.39	1.93	4.52	3.27	1.53	3.93	3.18	2.52	Median (range) 3.18 (1.53–4.52)
Desferioxamin therapy (Yes/No)	Yes	Yes	No	No	No	No	Yes	Yes	Yes	Yes	Yes	7 Yes/4 No
Ferritin (ng/ml)	676.5	387.2	544.0	289.9	1289.5	594.3	1490.4	503.5	254.4	1220.6	242	Median (range) 544 (242–1490.4)
Liver iron concentration (LIC, mg/g)	2.3	1.1	–	1.3	11.5	4.3	7.7	1.3	1.1	1.0	2.71	Median (range) 1.8 (1–11.5)
Cardiac iron concentration (CIC, T2* ms)	37	46	–	45	46	44	45	42	44	34	42	Median (range) 44 (34–46)
Hypogonadism (Yes/No)	Yes	Yes	Yes	Yes	Yes	No	Yes	Yes	No	Yes	Yes	9 Yes/2 No
Hypothyroidism (Yes/No)	Yes	No	No	No	No	No	Yes	No	No	No	No	2 Yes/9 No
Hypoparathyroidism (Yes/No)	Yes	No	No	No	No	No	No	No	No	No	No	1 Yes/10 No
Hyposurrenalism (Yes/No)	No	No	No	No	No	No	No	No	No	No	No	0 Yes/11 No
Growth hormone deficiency (Yes/No)^a^	–	–	No	No	–	–	No	–	No	No	Yes	1 Yes/5 No
Diabetes mellitus (%)	Yes	No	No	No	No	No	No	No	Yes	No	Yes	3 Yes/8 No
Zinc (µg/dl, normal range 70–120)	124.0	103.4	102.0	101.5	87.2	102.0	104.5	104.2	128.5	146.6	62.00	Median (range) 103.4 (62–146.6)
PTH (pg/ml)	13	53	31	27	24	22	32	22	28	40	26	Median (range) 27 (13–53)
25(OH)D (ng/ml)	12	30	10	25	21	43	22	20	30	24	22	Median (range) 22 (10–43)
Glomerular filtration rate (ml/min)	90	90	90	90	90	90	90	90	90	79	90	Median (range) 90 (79–90)

^a^54.5% of subjects was tested for GH deficiency

All patients had regular transfusion therapy, and hemoglobin levels were maintained within the target level (mean annual hemoglobin pre-transfusion levels: 9.61 ± 0.55 g/dl; target levels: 9.0–10.5 g/dl). The degree of erythroid proliferation was 3.00 ± 0.89 (acceptable values < 3). All patients were on chelation therapy to keep ferritin levels within the target level (mean annual ferritin levels: 681.12 ± 445.92 ng/ml; target levels: 500–1000 ng/ml). None of them had severe liver iron overload or cardiac iron overload. 45% had a slight or medium liver iron overload (normal LIC < 2; severe liver iron overload if LIC > 14; cardiac iron overload if T2* < 20). Hypogonadal patients continued their hormone replacement therapy (2 females and 5 males). Two hypogonadal females in fertile age refused replacement treatment (n° 3 and 10). Two patients, one male (n° 9) and one female (n° 6), presented no gonadal axis impairment. In addition, patients with hypothyroidism, hypoparathyroidism and diabetes mellitus were appropriately controlled with specific therapy.

**Fig. 1:**
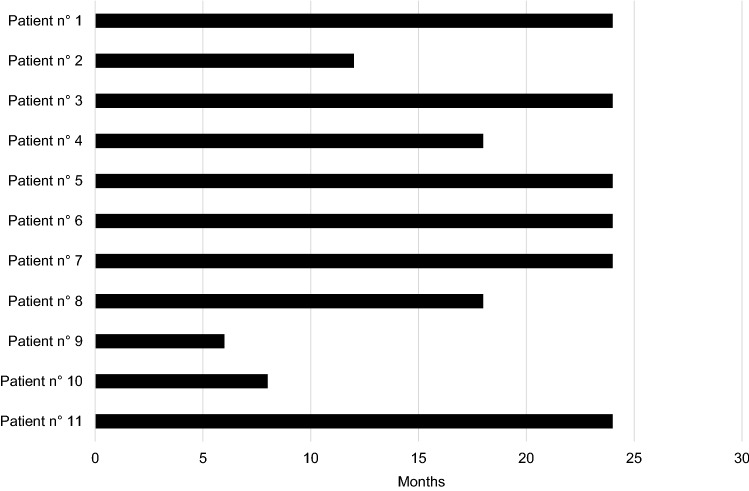
Teriparatide treatment duration

TP treatment period is shown in Fig. 1. The mean duration of TP treatment was 18.73 ± 7 months. Indeed, 9 and 6 subjects completed 12 months and 24 months of TP therapy, respectively. Two patients withdrew therapy due to poor compliance (one after 6 months and the other after 18 months), while 3 subjects withdrew therapy due to side effects development (fever and osteo-muscular pain) after 8, 12 and 18 months.

### Efficacy

#### Bone Mineral Density

**Fig. 2:**
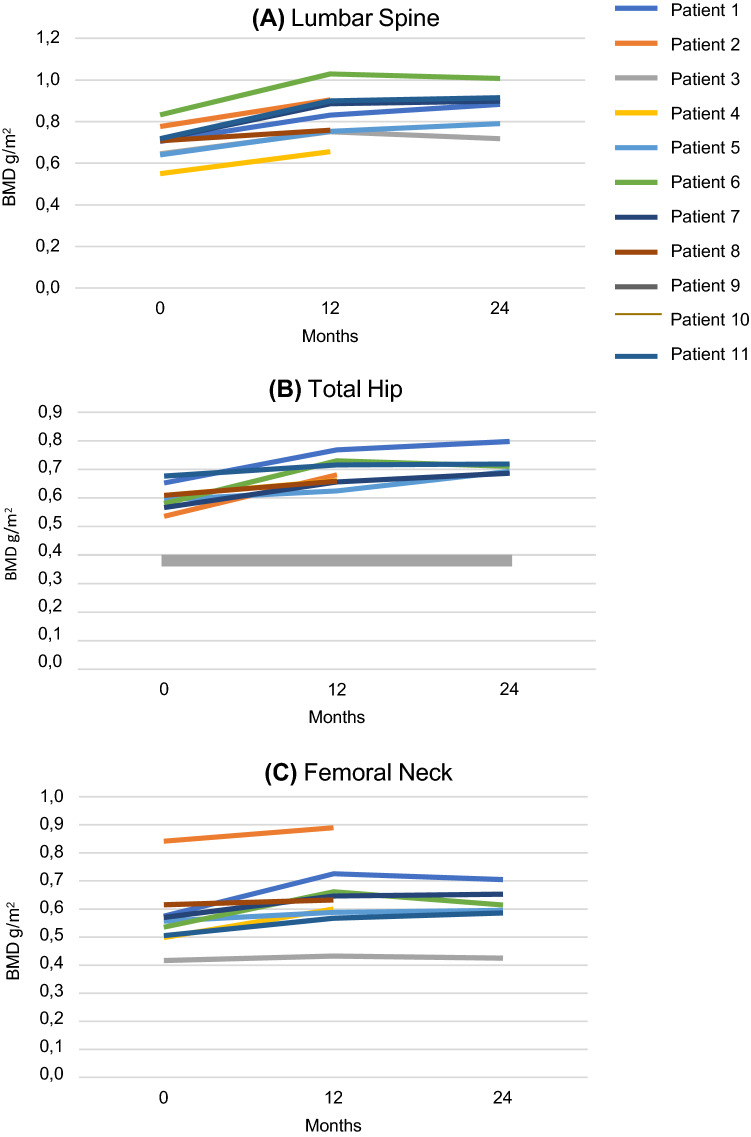
Lumbar spine (**A**), total hip (**B**) and femoral neck (**C**) BMD (g/cm^2^) in patients treated with teriparatide

Effects on BMD on different sites are reported in Fig. 2. At LS, BMD significantly increased after 12 (19%) and 24 months (22%). At TH, BMD increased by 13% after 12 months and by 14.2% after 24 months of therapy, reaching statistical significance only at 12 months. Finally, we observed a significant increase in BMD at the FN site both after 12 (12.65%) and after 24 months (12.8%).

#### Bone Turnover

Osteocalcin significantly increased after 12 (225%) and 24 months (54.2%) of therapy. Beta-cross laps levels showed a significant increase after 12 months (159%) and a non-significant increase after 24 months (141%). Mean 25(OH)D levels were 23.64 ± 8.88 ng/ml at baseline, 21.43 ± 8.85 ng/ml at 12 months and 24 ± 8.39 ng/ml at 24 months. No significant differences were found in 25(OH)D, serum and urinary calcium, serum and urinary phosphorus and serum alkaline phosphatase between baseline, 12 and 24 months of treatment.

#### Fractures

Before starting TP therapy, all 11 patients experienced at least one vertebral fracture, and 73% of them had two or more vertebral fractures. None had a previous femoral fracture, while 62.5% of patients had a history of one or more rib fractures. No new fractures were detected during TP treatment. After stopping TP treatment, four patients were switched to denosumab, six patients to bisphosphonates and one was kept under observation. After a mean follow-up of 6 ± 2 years, only one patient developed multiple vertebral fractures after 6 months from TP withdrawal and switching to denosumab.

#### Bone Pain

At baseline, all patients had bone pain except one. During TP treatment, 4 out of 9 patients who received 12 months of treatment (44.5%) reported pain improvement, two patients (22.2%) reported pain worsening, and three patients (33.3%) reported no variations. Overall, no significant differences were found in the mean VAS score between baseline (3.33 ± 2.96), 12 (2.89 ± 2.85) and 24 months (1.83 ± 2.64).

### Safety

Almost half of patients (5 out of 11) developed side effects. In particular, five patients reported bone pain (50%), four muscle pain (40%), and one fever (10%) after drug injection. In addition, three patients withdrew therapy due to side effects, respectively at 8, 12 and 18 months. Moreover, one patient temporarily discontinued therapy due to bone pain and two patients required chronic use of nonsteroidal anti-inflammatory drugs.

## Discussion

We described a series of TM patients on TP treatment for TM-associated osteoporosis. We observed a significant BMD improvement, especially at the LS site, and no new bone fractures occurred under treatment.

Osteoporosis is a significant cause of morbidity in TM patients, even in those subjects regularly treated with transfusion, chelation and hormonal replacement therapy [2, 4]. BP represent a reasonable treatment choice due to the evidence of an increased osteoclast function in TM associated osteoporosis. However, BP long-term efficacy and safety in TM have not been thoroughly established so far [7]. Usually, an increased osteoclastic function should be balanced by a corresponding increased bone formation. However, TM patients show impaired bone remodeling as supported by previous histomorphometric studies [5, 12, 17]. Many factors seem to contribute to TM-associated osteoporosis. The Wnt/ß-catenin pathway has been suggested to have a role in promoting osteoblast cell line differentiation, maturation and survival. As a consequence, in TM population the Wnt/ß-catenin pathway could be involved in the pathogenesis of osteoporosis and negative modulators of this signaling system, such as Dickkopf-1 and sclerostin, have also been associated with BMD in TM patients [5, 18, 19]. Low IGF-1 levels could be another factor that could be implicated in TM-associated osteoporosis development. Low IGF-1 may be responsible for decreased osteoblast proliferation and bone matrix formation [20]. At the same time, some oral iron chelation therapies might represent an impacting factor inhibiting DNA synthesis, collagen formation and osteoblast precursor differentiation [21, 22]. In this scenario, the choice of an anabolic agent promoting osteoblast activity could be reasonable. TP is the only anabolic agent currently approved to treat postmenopausal, male and glucocorticoid-induced osteoporosis in Italy. TP is a recombinant fragment of human parathyroid hormone (PTH) consisting of its first amino(N)-terminal 34 amino acids. When administered intermittently, it exerts a bone anabolic function by binding the PTH type 1 receptors expressed on osteoblasts and osteocytes. TP seems to upregulate transcriptional expression of pro-osteoblastogenic growth factors like IGF-1 and fibroblast growth factor 2 (FGF2). In addition, it modulates the Wnt/ß-catenin osteoanabolic signaling by down-regulating the synthesis of sclerostin [12, 23, 24]. In-vivo studies demonstrated that in rat after few days of intermittent TP treatment, resting bone lining cells turned into mature osteoblasts cells [25, 26]. In no-TM population, TP treatment has been shown to increase lumbar spine BMD by 10% after 18 months of therapy and to reduce vertebral and non-vertebral fracture risk by 65% and 53%, respectively, after 21 months of therapy [12, 13, 27]. A recent meta-analysis comparing TP with placebo showed a decreased vertebral and no-vertebral fractures risk of 74% and 39%, respectively [28, 29]. Moreover, TP treatment was demonstrated to reduce frequency and severity of back pain [13, 30]. Despite evidence of safety and clinical efficacy in general population, data on TP treatment in TM population are still sparse and several issues are still open such as the possibility to worsen any concomitant hypercalciuria or lead to the development of extramedullary hematopoiesis [31]. To our knowledge, TP treatment in TM-associated osteoporosis is documented in only two case reports. Trotta et al. [32] described a case of a TM 43 years old woman affected by osteoporotic multiple vertebral fractures, despite BP treatment. She was subsequently switched to TP for 18 months. At the end of the treatment, lumbar spine and femoral BMD increased by 16.3% and 30.5%, respectively. No new fracture was detected. Similarly, Tournis et al. [33] reported a case of a 34 years old TM male patient having multiple osteoporotic vertebral fractures not satisfactorily treated with alendronate. TP was started, and after six months there was a substantial decrease in FN and TH BMD, but subsequently, BMD started increasing slowly with a substantial increase (9.3% al FN and 9.8% at TH) between 12 and 18 months of treatment. Lumbar BMD was not evaluated because of a previous vertebroplasty procedure. No new fractures were detected. The patient was then switched to alendronate for the following four years, but a new fracture occurred. A second trial of TP treatment was started, resulting in a BMD increase of 3.4% in FN and TH at six months, of 10% in FN and of 5.3% in TH at 12 months. In both case reports, TP was well tolerated. The patient reported by Trotta et al. also showed decreased back pain and an improved quality of life. 

In our series, we observed a significant BMD increase at LS, TH and FN after 12 months of TP treatment; this finding was confirmed at LS and FN sites after 24 months of TP treatment. BMD improvement was more significant at LS and during the first year of therapy. Similarly, we observed a significant increase in osteocalcin and beta-crosslaps, supporting the activation of bone turnover. No patient experienced a new fracture during TP treatment. TP demonstrated a limited efficacy only in one hypogonadal patient not satisfactorily replaced with concomitant testosterone therapy (subject n°3), confirming the importance of careful management of TM-related endocrine comorbidities.

Therefore, our results are in line with data of the two case reports described above [32, 33], supporting TP efficacy in TM patients and the possibility of a sequential multi-drug approach. An Italian group of experts considers TP as a possible second-line therapy in severe TM-associated osteoporosis with multiple fractures after prolonged BP treatment or when patients are non-responders to BP therapy or present severe BP side effects [6, 34]. Moreover, considering the consistent BMD increase mainly during the first year of therapy and the need for lifelong osteoporosis management in TM population, re-treatment with TP after the first 12 months of therapy may be proposed, as suggested by Tournis et al. [33]. Furthermore, TM-associated osteoporosis can be considered a condition at high-risk of fractures, based on its multifactorial pathogenesis, which hinders attainment of an optimal peak bone mass, and compromises anabolic bone function. Therefore, TM patients might benefit by a first-line treatment with TP aiming to increase bone mass, followed by antiresorptive agents. However, no supporting data for this approach are available so far.

All osteoporosis treatment regimens require calcium and 25(OH)D supplementation to improve mineralization [29]. In our population, mean 25(OH)D levels were < 30 ng/ml and this might have impaired therapeutic efficacy. However, the optimal serum 25(OH)D levels to ensure the best TM bone turnover have not been defined yet. In the TM population, a higher rate of 25(OH)D deficiency and insufficiency is described as compared to no TM patients, as well as a negative and non-linear relationship between BMD and 25(OH)D levels [4, 35]. Vogiatzi et al. demonstrated that the inverse relationship between 25(OH)D and BMD reaches a plateau for levels > 15 ng/ml [36].

Regarding safety, in our study 50% of patients reported bone pain, 40% muscular pain and 3 out of 11 patients withdrew therapy due to side effects. No significant difference was found in the mean VAS score between baseline, 12 and 24 months, even if a wide heterogeneity was observed. Three patients reported bone pain improvement, four patients a worsening and one patient no variations. These data are in contrast with what reported in “no TM” population. In TP pivotal study, drug withdrawal due to adverse events was registered in 6% of women treated with 20 μg of TP and 11% treated with 40 μg [12]. An Italian observational multicenter real-life study reported high adherence to TP treatment and excellent tolerance to the drug and described only minimal adverse events, which did not lead to discontinuation of therapy [37]. Furthermore, TP treatment has shown a reduction in frequency and severity of back pain in “no TM” population [13, 27]. The mechanism of reduction in back pain in osteoporotic patients treated with TP is still not clear. It may be linked to the decreased risk of new painful vertebral fractures or to the stabilization of pre-existing fractures [13, 27]. Pathogenesis of bone pain in TM is complex, and this could explain the higher frequency of bone pain in TM patients treated with TP compared to “no TM” population [35, 38]. The most common site is the lower back, but frequently patients report pain in other sites, such as upper and lower limbs and head [7]. In addition to vertebral deformities, bone changes due to the expansion of bone marrow could play a crucial role in the pathogenesis of bone pain. Some adults experienced increasing pain at the end of transfusion cycles when hemoglobin level drops, meanwhile others report lower pain after transfusion [30, 39]. However, data are conflicting, and no differences have been found in terms of hemoglobin levels, chelation regimen, iron overload, history of fractures, BMD or BP use comparing patients who report pain to those who do not [5, 30, 40]. Therefore, we can hypothesize a relationship between bone marrow expansion that interrupts bone formation and TP mechanism of action, which promotes bone formation. However, this hypothesis needs confirmation. In our population, we did not find any difference between patients who reported pain compared to those who did not regarding hemoglobin levels, transferrin soluble receptor, degree of erythroid proliferation, or iron overload markers. Furthermore, TP requires a daily injection affecting patients compliance.

A limit of our study is the small sample size, even if it should be noted that TM is a rare disease. Furthermore, long-term safety information is lacking and this could not allow us to collect data about bone marrow expansion. However, to our knowledge, this is the largest case series describing TP treatment in TM-associated osteoporosis. Our results support the anabolic efficacy of TP in increasing BMD also in TM population opening the possibility of a new tailored approach to the life-long management of TM-associated osteoporosis. Indeed, anabolic treatment as first-line therapy could represent a reasonable choice in TM patients who often present with low BMD and high fracture risks due to many predisposing factors, as previously described. BMD optimization might be pursued before starting antiresorptive treatment in order to save and preserve BMD gain lifelong. Further longitudinal and prospective studies are needed to support this hypothesis.

## Conclusion

In conclusion, TP might be an effective treatment for TM-associated osteoporosis since it improves BMD, especially at LS and prevents fragility fractures. TM patients seem to have a higher frequency of side effects, particularly muscle and bone pain under TP treatment, than “no TM” population. This phenomenon could be explained by the different and multifactorial pathogenesis of osteoporosis and bone pain in TM. Further studies are required to confirm the long-term efficacy, safety and treatment schedule of TP therapy for TM patients and to explain the physiopathological mechanisms, especially concerning bone pain. Additional studies are also needed to improve lifelong management of TM-associated osteoporosis and to plan sequential treatments.
